# Supplementary material for: Incidence and Predictors of Cardiac Implantable Electronic Devices Malfunction with Radiotherapy Treatment
Source: J Clin Med. 2022 Oct 27;11(21):6329. doi: 10.3390/jcm11216329 (PMC9654752; doi:10.3390/jcm11216329)
Supplement: Supplementary file 1 [file jcm-11-06329-s001.zip › jcm-1901554-supplementary.pdf]

Table S1. Recorded device malfunction during radiotherapy. CIED, cardiac implantable electronic device; CRT–P, cardiac resynchronization therapy pacemaker; CRT–D; cardiac resynchronization therapy defibrillator; DC PPM, dual chamber permanent pacemaker; Gy, gray, ICD, implantable cardioverter defibrillator; MV, megavolt; RT, radiotherapy; V, volt.

Device type	Manufacturer	Anatomical Area of Radiation	Cumulative RT Dose/Dose per Fraction, Gy	Beam Type	Beam Energy, MV	Description
DC PPM	Medtronic	Thorax	60/17	Photon	13	Increased RV impedance of 1102 Ω 6 months post last RT fraction. RT was completed and no evidence of lead fracture was found.
DC PPM	Medtronic	Thorax	45/14	Photon	14	Asymptomatic transient ventricular undersensing. RT was completed and the device successfully reprogrammed
DC PPM	St Jude	Head and neck	50/16	Photon	10	Unexpected decline in battery by 5% a month after the last RT fraction. Increase in frequency of CIED interrogation.
DC PPM	Boston Scientific	Thorax	77/26	N/A	N/A	Reduced pacing lead output from 7.5 to 4.0 V after 3rd palliative RT fraction. RT was completed and the patient received no further action as per patient's wishes.
DC PPM	Boston Scientific	Abdomen and pelvis	47/14	Photon	12	Battery depletion 6 months post last RT dose. An urgent CIED generator replacement was performed
CRT–P	Medtronic	Thorax	50/11	Photon	12	Unexpected decline in battery by 10% 2 months post RT. Elective generator change was scheduled.
ICD	Medtronic	Thorax	35/10	Photon	14	Asymptomatic short-lived inappropriate tachycardia sensing during RT. No shock

						was delivered, and RT course was completed
ICD	Medtronic	Thorax	30/15	Photon	10	Partial reset after 3rd fraction of RT. RT was completed and no further action was required.
ICD	Medtronic	Head and neck	32/12	Photon	17	Electrical reset and trend data error 2 months post RT. The device was successfully reprogrammed by the CIED technician.
ICD	St Jude	Thorax	40/13	Photon	15	Asymptomatic increase in sensing rate during RT. No shock was delivered and the ICD was successfully reprogrammed
ICD	St Jude	Abdomen and pelvis	40/13	Photon	11	Partial reset after 3rd fraction of RT. RT was completed and no further action was required.
ICD	St Jude	Thorax	30/10	Photon	18	Asymptomatic short-lived inappropriate tachycardia sensing during RT. No shock was delivered and the RT course was completed
ICD	Boston Scientific	Thorax	32/12	Photon	16	Asymptomatic short-lived inappropriate tachycardia sensing during RT. No shock was delivered, and the RT course was completed
ICD	Biotronik	Head and neck	35/11	Photon	17	Partial reset after 2nd fraction of RT. No further action was required, and the patient was cleared for the next RT fraction
CRT–D	Medtronic	Thorax	44/15	Photon	16	Asymptomatic short-lived inappropriate tachycardia sensing.

						No shock was delivered and the RT course was completed
CRT–D	Medtronic	Thorax	20/10	Photon	12	Electrical reset with loss of stored data during 2nd RT fraction. Device reprogrammed by CIED technician
CRT–D	Medtronic	Thorax	48/15	Photon	11	Battery depletion 4 months post last RT fraction. An urgent CIED generator replacement was performed
